# Developmental Expression Patterns of miRNA in *Mythimna separata* Walker (Lepidotera: Noctuidae)

**DOI:** 10.3390/genes16020234

**Published:** 2025-02-19

**Authors:** Yuhan Liu, Huiman Tian, Shaoqiu Ren, Xiulin Chen, Kun Luo, Guangwei Li, Boliao Li

**Affiliations:** Key Laboratory of Applied Ecology of Loess Plateau, College of Life Science, Yan’an University, Yan’an 716000, China; 13060498188@163.com (Y.L.); tianhuiman2580@126.com (H.T.); 17710451579@163.com (S.R.); xiulin@yau.edu.cn (X.C.); luok@yau.edu.cn (K.L.); liguangwei@yau.edu.cn (G.L.)

**Keywords:** migratory insect, armyworm, development, small RNA sequencing

## Abstract

Background/Objectives: miRNAs are a family of single-stranded non-coding RNAs that regulate gene expression by targeting messenger RNAs (mRNAs) for suppression, with an average length of 22 nt. The oriental armyworm, *Mythimna separata* Walker, is a pest insect with long-distance migratory capability, which causes severe loss of grains and pastures in Eastern Asia, Southeastern Asia, and Oceania. This study aims to elucidate the post-transcriptional regulatory mechanisms of miRNAs in the development of this pest. Methods: We carried out small RNA sequencing on samples from eggs, third instar larvae, pre-pupae, pupae, and adults. Results: A total of 400 miRNAs were identified, among which 40 were known and 360 were novel miRNAs. Dynamic trend analysis of miRNAs revealed that 199 miRNAs were highly expressed in eggs (profile 12), while 173 miRNAs were highly expressed in both eggs and pupae (profile 13). The results of differential expression analysis of miRNAs (DEmiR) revealed that 75 miRNAs were significantly more abundant in eggs compared to other developmental stages. Furthermore, more up-regulated miRNAs were observed than down-regulated miRNAs in adults relative to 3rd instar larvae, pre-pupae, and pupae. The core genes for miRNA biosynthesis—*Pasha*, *Dicer1*, and *Ago1*—were highly expressed in eggs but poorly expressed in 3rd instar larvae. KEGG enrichment analyses indicated that several genes in the pentose and glucuronate interconversion pathway, as well as the fructose and mannose metabolism pathway, were regulated by DEmiRs. Conclusions: DEmiRNAs targeted most genes of *M. separata*, resulting in a complex miRNA–mRNA regulation mode.

## 1. Introduction

miRNAs are a family of single-strand non-coding RNAs, typically 18–26 nt, which are commonly found in eukaryote species [1]. Most miRNAs in animals bind to the 3′ UTR of mRNAs, where they degrade the mRNAs or inhibit protein translation, depressing the target mRNA expression. Some miRNAs also bind to the 5′UTR or CDS to regulate gene expression [2,3]. High-throughput sequencing has been extensively employed to identify miRNAs across insect species, including *Bombyx mori* [4,5], *Plutella xylostella* [6], *Ostrinia furnacalis* [7], *Spodoptera frugiperda* [8], *Grapholita molesta* [9], *Bactrocera dorsalis* [10], *Apis cerana* [11], *Bemisia tabaci* [12], and *Galeruca daurica* [13]. miRNAs are involved in nearly all biological processes in insects, such as cell division and apoptosis [14], metamorphosis [15,16,17,18,19], integument formation [20], physiological metabolism [14,21,22], immunity [23], and reproductive system maturation [24].

miRNAs in animals are initially transcribed as pri-miRNA, which then encompasses one or more hairpin-structured pre-miRNAs with the catalysis of Drosha and Pasha within the nucleus. Subsequently, the pre-miRNAs are cleaved by Dicer-1 combined with Loquacious into an imperfectly paired duplex. Finally, one strand of duplex is kept while the other one is degraded. The mature miRNA is incorporated into Ago1 to form miRISC, leading to a repression of translation of mRNA or its degradation [3,25].

The oriental armyworm, *M. separata* Walker (Lepidoptera: Noctuidae), also known as the northern armyworm, is a destructive agriculture pest that damages crops and pastures in Poaceae [26,27], which is distributed in the eastern part of Asia and Oceania [28]. In China, *M. separata* overwinter in the south and annually undergo four distinct large-scale migrations in eastern China, including two early northward and two late southward [29,30]. Its huge food intake in late instar larvae, excellent flying capacity, and huge fecundity of adults make the outbreak of this pest difficult to predict [31].

dsRNA interference has been frequently used in functional research of genes on *M. separata* [32,33,34]. However, the stability of dsRNA interferences in Lepidoptera has been proven to vary depending on species, tissues, gut environment, and gene function [35]. Interference using miRNA offers an alternative method for investigating gene functions. miRNAs have been characterized across various insect orders; however, there is an absence of published research documenting miRNAs in *M. separata*. In this study, we screened conserved and novel miRNAs from *M. separata* across different developmental stages and predicted their target genes based on small RNA libraries. This research provides a valuable resource to elucidate the regulatory roles of miRNAs at the post-transcription level.

## 2. Materials and Methods

### 2.1. Insect Rearing and Sample Collection

The larvae of *M. separata* were originally collected from Xingping, China, in 2014 (108.42 °E, 34.28 °N) and then were continuously reared in the laboratory. Larvae of *M. separata* were fed with fresh wheat leaves, with a temperature of (25 ± 1) °C, a relative humidity (RH) of (65 ± 10)%, and a photoperiod of 14 L:10 D. Before 4th instar larvae, they were reared in collection. Then, they were fed, with five larvae in every 400 mL plastic cup, which were covered with plastic wrap with fine holes. Pre-pupae were transferred to peat soil for pupation. Each pair of emerged adults was fed with 5% honey solution in a 400 mL plastic cup. The insect samples were killed by liquid nitrogen and stored at −80 °C until the extraction of total RNA.

### 2.2. Small RNA Library Preparation and Sequencing

Each RNA sample was extracted from about 200 eggs, six larvae in 3rd instar, three pre-pupae, three pupae, and two pairs of adults, using TRIzol reagent (Takara, Dalian, China). Three biological repeats were set. The purity and concentration of RNA samples were examined by 1% agarose gel electrophoresis. The total RNA quantity and purity were assessed using a Bioanalyzer 2100 and RNA 1000 Nano Lab Chip Kit (Agilent, Santa Clara, CA, USA), ensuring an RNA integrity number greater than 7.0. For library preparation, the Illumina TruSeq Small RNA Sample Prep kit (Illumina, San Diego, CA, USA) was utilized following the manufacturer’s instructions. High-throughput RNA sequencing was carried out on the Illumina HiSeq4000 sequencer platform in PE150 mode (Biomarker Technologies, Beijing, China).

### 2.3. Small RNA Data Analysis

Raw reads were trimmed using TrimGalore (v. 0.6.10) [36], a wrapper around Cutadapt and FastQC, to remove low-quality reads, short sequences (<18 nt), and adaptors. Using Bowtie (v. 1.2.3) [37], the clean reads were aligned with Rfam (14.0) [38] to filter ribosomal RNA (rRNA), small cytoplasmic RNA (scRNA), small nuclear RNA (snRNA), small nucleolar RNA (snoRNA), and transfer RNA (tRNA). Subsequently, the residual reads were mapped to the *M. separata* genome to find exons, introns, and repeat sequences (NCBI assembly: GCA_029852925.1) [39]. Finally, known miRNAs were identified by mapping to miRBase (v. 21) using blastn (v. 2.14.0) [40], and novel miRNAs were predicted using miRDeep 2 (v2.0.1.3) [41].

### 2.4. Target Gene Annotation of miRNA

miRanda (v.3.3a) [42], RNAhybrid (v. 2.1.2) [43], and targetscan (v. 7.0) [44] were used for the prediction of target genes of miRNAs (1000 nt downstream of target genes). We set the seed length ≥ 6 for targetscan; the energy for predicted interactions was <−15 kcal/mol for miRanda and −20 kcal/mol for RNAhybrid. The target genes were annotated against NR (date: 202306), Uniprot-SwissProt (date: 202401), and Uniprot-TrEMBL (date: 202401) using diamond (v. 2.1.8) [45], and to KEGG and Pfam (Protein family) databases using eggNOG-mapper v2 [46,47].

### 2.5. Differential Expression Analysis of miRNAs (DEmiRs) and Enrichment Analysis of Target Genes Responding to DEmiRs

The dynamic trend analysis of miRNAs was performed on the OmicShare tools (www.omicshare.com/tools) (accessed on 12 September 2024) [48]. The differential expression analyses of miRNAs from different developmental stages were conducted using the *Perl* script *run_DE_analysis.pl* wrapped in Trinity (v. 2.14.0) [49], which employs the *DESeq2* package (v. 1.14.1) method [50] with default parameters. Low-expressed miRNAs were filtered using the criterion of *rowSums* (*cpm* (*rnaseqMatrix*) > 1) ≥ 3. The criteria to determine the DEmiRs were set as |log2FoldChange| ≥ 1 and *padj* < 0.01). Then, KEGG enrichment analyses for the target genes of DEmiRs were performed using the R package clusterProfiler (v. 4.10.0) [51]. Pearson correlation analysis, PCA, and hierarchical analysis were performed by R (v. 4.4.2).

### 2.6. RT-qPCR

For miRNA quantification, we used the miRNA 1st Strand cDNA Synthesis Kit (by tailing A) (Vazyme, Nanjing, China) to generate cDNA of miRNAs. The RT-qPCR was performed, and the 20 μL RT-qPCR system contained 2 μL of diluted cDNA, 10 μL of 2 × ChamQ Universal SYBR qPCR Master Mix (Vazyme, Nanjing, China), 0.4 μL of each specific primer (10 μM), 0.4 μL of Universal reverse Q primer (10 μM) (Vazyme, Nanjing, China), and 7.2 μL RNase-free water. RT-qPCR was conducted using a StepOnePlusTM Real-Time PCR System (ThermoFisher Scientific, Waltham, MA, USA) under the following conditions: 95 °C for 5 min, followed by 40 cycles of 95 °C for 10 s and 60 °C for 30 s. We set three independent biological replicates and three technical replicates to keep repeatability. The quantification of miRNAs was calculated by normalizing to *small nuclear RNA U6* (*U6*) [10,19,52,53].

For mRNA quantification of three miRNA biosynthesis core genes, the cDNA was prepared using HiScript^®^ II Q RT SuperMix for qPCR (+gDNA wiper) (Vazyme, Nanjing, China). qPCR was carried out using ChamQ Blue Universal SYBR qPCR Master Mix (Vazyme, Nanjing, China) according to the instruction manuscript on a StepOnePlus^TM^ Real-Time PCR System (ThermoFisher Scientific, Waltham, MA, USA). *Ribosomal protein S13* (*rpS13*) and elongation factor 1α (*EF-1α*) were selected as internal control genes [54]. The primers used for RT-qPCR are listed in Table S1.

The 2^−ΔΔCt^ relative quantification method was used to analyze the data for miRNAs and mRNAs, with three biological replicates for each treatment [55]. One-way ANOVA followed by Tukey’s multiple comparison were conducted to examine the statistical expression of miRNAs and miRNA biosynthesis core genes on R (v. 4.4.2).

## 3. Results

### 3.1. Summary of Small RNA Sequencing

**Figure 1:**
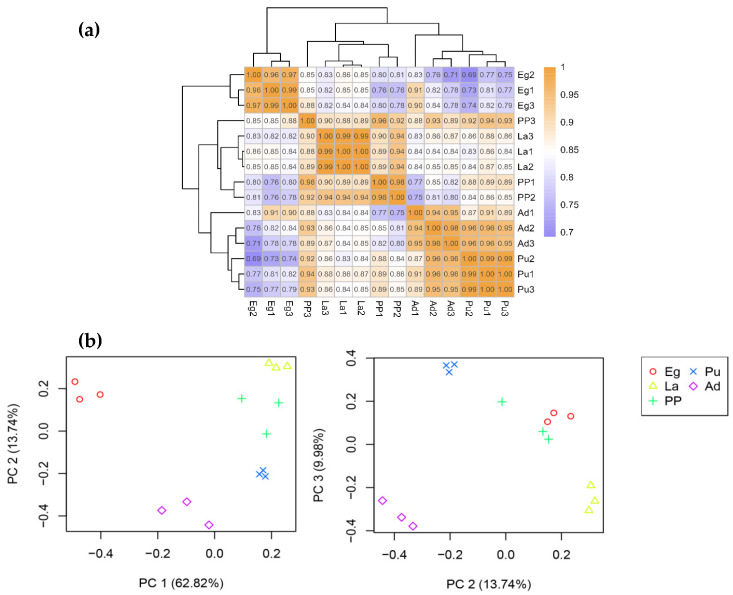
The repeatability of miRNA samples from five developmental stages of *M. separata*. (**a**) Correlation analysis of miRNA read counts from 15 samples after mRNA, rRNA, snRNA, snoRNA, tRNA, and repeat sequences were filtered. The color spectrum, ranging from purple to orange, represents Pearson correlation coefficients. (**b**) PCA (principal component analysis of 15 miRNA samples among five groups. Eg, Egg; La, 3rd instar larvae; PP, Pre-pupae; Pu, Pupae; Ad, adult.

Fifteen small RNA libraries were constructed and sequenced to identify miRNAs in *M. separata*. A total of 18,557,750, 18,064,725, 16,527,980, 15,122,711, and 16,302,273 clean reads were produced in the libraries of egg, 3rd instar larvae, pre-pupae, pupae, and adults, respectively. Q20 was more than 99.34%, and Q30 ranged from 96.93 to 97.91% (Table S2). Mapping of miRNAs to the *M. separata* reference genomic sequences ranged between 78.60% and 99.52% of the reads from 15 libraries (Table S2). Pearson’s correlation analysis (Figure 1a), PCA (Figure 1b), and clustering analysis (Figure S1) showed a strong correlation between each sample within the developmental stage, ensuring the data reliability for the following analyses. After filtering out mRNA, rRNA, snRNA, snoRNA, tRNA, and repeat sequences, the remaining reads were used to identify miRNAs (Table S3).

### 3.2. miRNA Identification and Differential Expression Analysis

**Figure 2:**
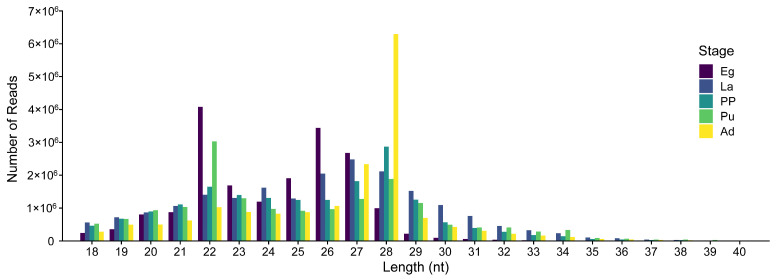
Length distribution and abundance of miRNAs from *M. separata* in deferent developmental stages. Eg, Egg; La, 3rd instar larvae; PP, Pre-pupae; Pu, Pupae; Ad, adult.

A total of 400 miRNAs were identified, comprising 40 known and 360 novel across 15 libraries (Table S4). The lengths of miRNA predominantly ranged from 22 nt to 28 nt. Specifically, 22 nt was dominant in the Eg group, while 27 nt was dominant in the La group, and 28 nt was dominant in the PP, Pu, and Ad groups (Figure 2). These findings align with the typical length characteristics of miRNA length.

**Figure 3:**
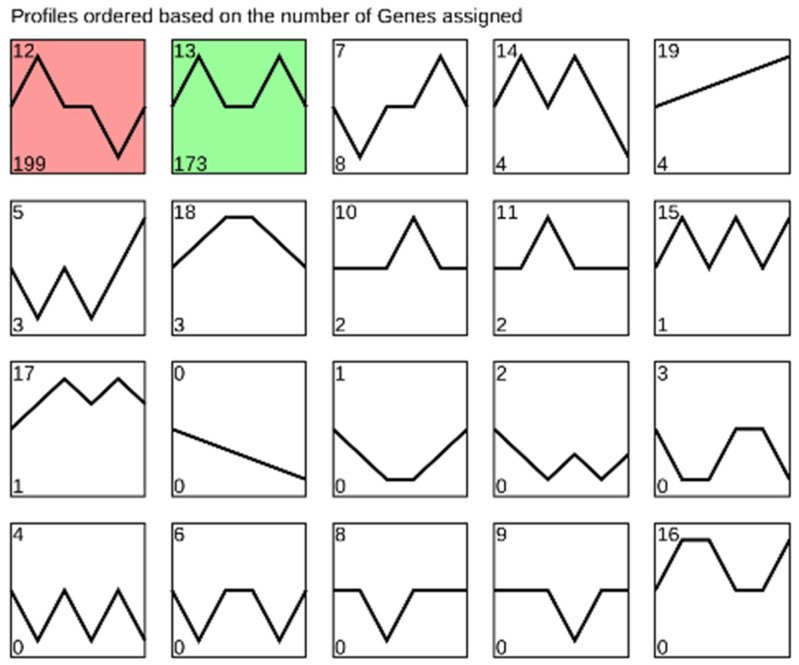
Dynamic trend analysis of miRNAs from *M. separata*. The significant clustered profiles were colored.

**Figure 4:**
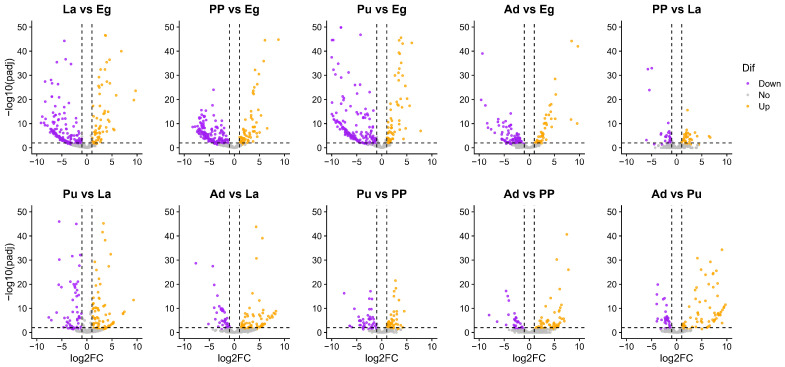
Volcano plots of differentially expressed (DE) miRNAs between any two of five developmental stages of *M. separata* (|log2FoldChange| > 1 and *padj* < 0.01). Purple dots indicate significant down-regulated miRNAs, orange dots indicate up-regulated miRNAs, and grey dots indicate miRNAs with no significant difference.

The results of dynamic trend analysis of miRNAs from *M. separata* across five developmental stages showed that the majority of miRNAs were categorized into cluster 12 (199 miRNAs highly expressed in eggs) and cluster 13 (173 miRNAs highly expressed in eggs and pupae) (Figure 3). Differential expression analysis of miRNAs across the developmental stages was performed using a cutoff of |log2FoldChange| > 1 and *padj* < 0.01. The results showed that 75 out of 400 miRNAs exhibited significantly higher expression in eggs compared to all the other developmental stages (Figure 4, Table S5). In addition, more up-regulated than down-regulated miRNAs were observed in adults when compared to miRNAs from 3rd instar larvae, pre-pupae, and pupae (Figure 4, Table S5).

### 3.3. RT-qPCR Analysis of miRNA Biosynthesis Core Genes and qPCR Validation of miRNAs

**Figure 5:**
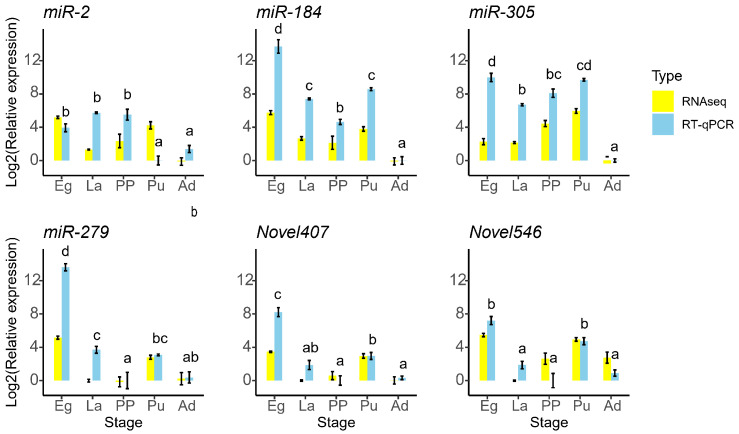
Relative expression of miRNAs from *M. separata* in different developmental stages. The yellow vertical bars represent the log2-transformed fold change in miRNA expression for each stage compared to the mean expression level of the lowest expressed stage based on small RNA sequencing. Bars represent the *means* ± *SE* (*n* = 3). Different lowercase letters above the sky blue vertical bars represent significantly different expression among different developmental stages that were analyzed by Tukey’s multiple comparison based on RT-qPCR (*p* < 0.05). The relative expression of each miRNA was normalized to *U6*.

**Figure 6:**
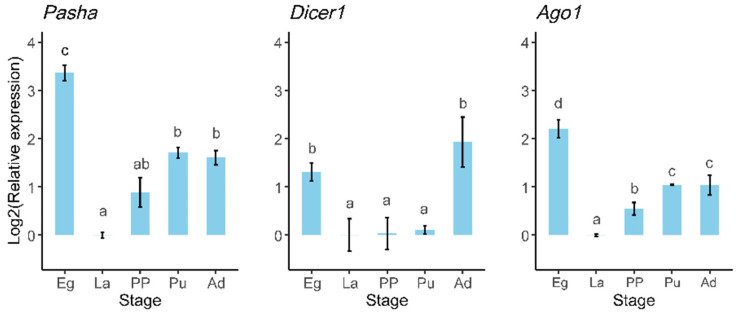
Relative expression of miRNA biosynthesis core genes in *M. separata* in different developmental stages. Bars represent the *means* ± *SE* (*n* = 3). Different lowercase letters above the vertical bar represent significantly different expression among different developmental stages that were analyzed by Tukey’s multiple comparison. The relative expression of each gene was normalized to the geometrical mean of *ribosomal protein S13* (*rpS13*) and *elongation factor 1α* (*EF-1α*).

Six DEmiRNAs were selected for validation via RT-qPCR, showing that *miR-184*, *miR-305*, *miR-279*, and *Novel407* exhibited significantly higher expression levels in eggs compared to the other developmental stages. Specifically, *miR-184* and *miR-305* displayed the lowest expression in adults, whereas *miR-279*, *Novel407*, and *Novel546* exhibited the lowest expression levels during the pre-pupal stage (Figure 5). These six miRNAs showed similar trends of expression in different stages between the results of small RNA sequencing and RT-qPCR, indicating the credibility of small RNA sequencing. Furthermore, the expression levels of *Pasha* and *Ago1* were significantly elevated in eggs relative to the other stages but were notably reduced in 3rd instar larvae. Additionally, *Dicer1* expression was higher in eggs and adults than in the other three stages (Figure 6).

### 3.4. Functional Analysis of miRNA Target Genes

**Figure 7:**
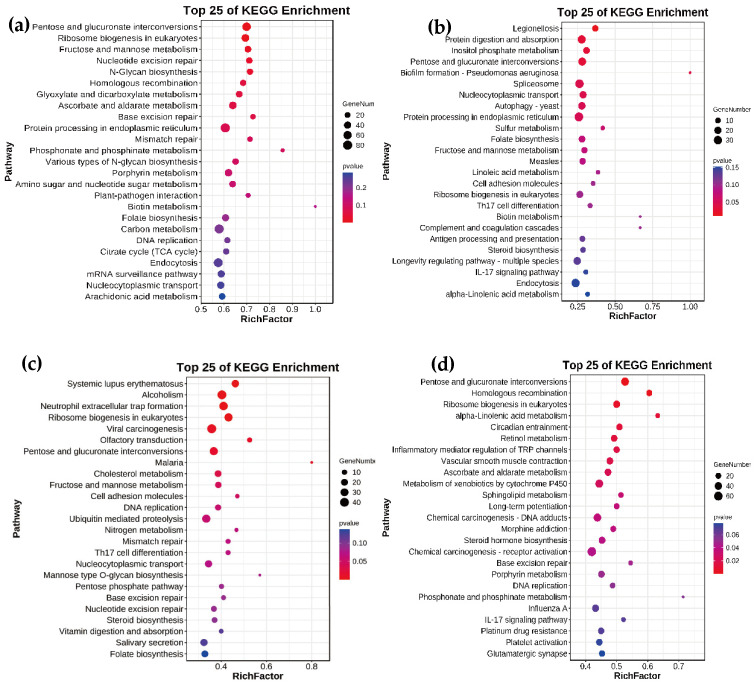
KEGG pathway enrichment analyses for target genes for differently expressed miRNAs from *M. separata* in various developmental stages. (**a**) 3rd instar larvae vs. eggs; (**b**) Pre-pupae vs. 3rd instar larvae; (**c**) Pupae vs. pre-pupae; (**d**) Adults vs. pupae.

To screen important pathways associated with development, we conducted KEGG enrichment analysis on the target mRNAs of miRNAs across various developmental stages (Figure 7). Five pathways were significantly enriched from target mRNAs of DEmiRs between eggs and 3rd instar larvae (*p* value < 0.05), including pentose and glucuronate interconversions, ribosome biogenesis in eukaryotes, fructose and mannose metabolism, nucleotide excision repair, and N-Glycan biosynthesis. Six pathways were significantly enriched from target mRNAs of DEmiRs between 3rd instar larvae and pre-pupae (*p* value < 0.05), including protein digestion and absorption, inositol phosphate metabolism, pentose and glucuronate interconversions, spliceosome, nucleocytoplasmic transport, and protein processing in endoplasmic reticulum. Additionally, six pathways were significantly enriched from target mRNAs of DEmiRs between pre-pupae and pupae (*p* value < 0.05), encompassing ribosome biogenesis in eukaryotes, olfactory transduction, pentose and glucuronate interconversions, cholesterol metabolism, fructose and mannose metabolism, and cell adhesion molecules. Finally, fifteen pathways were significantly enriched from target mRNAs of DEmiRs between pupae and adults.

## 4. Discussion

MicroRNAs (miRNAs) are essential for post-transcriptional regulation of gene expression. This regulation occurs when a duplex is formed between an miRNA and an mRNA CDS or 3′ UTR, leading to the degradation of target mRNAs or inhibition of the translational machinery [3,56,57]. In the present study, we analyzed miRNAs across five different developmental stages and identified several highly expressed miRNAs along with their potential targets, which may be related to the regulation of metamorphosis in *M. separata*. Previous studies have identified 354 miRNAs in *B. mori*, including 272 novel miRNAs [4], 462 miRNAs in *P. xylostella*, of which 174 were species-specific [6], and 108 known and 134 novel miRNAs in *Spodoptera litura* [58]. Additionally, 184 known and 65 novel miRNAs were identified in *B. dosalis* [10], and 99 known and 65 novel miRNAs were identified in *Anopheles sinensis* [59]. This study seems to be the first publication to identify miRNAs in *M. separata* using small RNA sequencing, identifying 400 miRNAs comprising 40 known and 360 novel miRNAs. The relatively low ratio of known miRNAs may partially reflect species-specific miRNA profiles. Another potential factor could be the limited representation of lepidopteran species in the Rfam database (v. 14.0), which currently includes only 137 miRNA precursors from *B. mori*, the only lepidopteron species in this database [38].

The results of dynamic trend analysis of miRNAs from *M. separata* in five developmental stages showed that most miRNAs were either highly expressed in eggs or displayed high expression in both eggs and pupae (Figure 3). While most studies on miRNA expression in holometabolous insects have primarily focused on their roles during the larval–pupal and pupal–adult transition, limited small RNA sequencing studies that include the egg stage suggest that abundant miRNAs in eggs and pupae seem common. This temporal pattern of miRNA expression has also been observed in *B. mori* and *P. xylostella* [4,6]. This trend likely contributes to the varying expression levels of miRNA biosynthesis core genes. For instance, in *Helicoperpa armigera*, *Pasha* exhibited significantly higher expression in male adults, followed by female adults, eggs, and 1st to 3rd instar larvae, with lower levels from 4th instar larvae to pupae. Similarly, *Dicer1* and *Ago1* showed significantly higher expression in eggs, 1st instar larvae, and adults compared to the other developmental stages. Additionally, *Drosha* and *loquacious* were richly expressed in eggs and 4th instar larvae [60]. In the current study, *M. separata* showed similar expression patterns for miRNA core genes. *Pasha*, *Dicer1*, and *Ago1* from *M. separata* were all highly expressed in eggs but showed minimal expression in 3rd instar larvae (Figure 6). This pattern may account for the observed abundance of miRNAs in eggs.

When conducting pairwise comparisons of DEmiRs across five developmental stages (La vs. Eg, PP vs. La, Pu vs. PP, Ad vs. Pu), a total of 143 down-regulated and 67 up-regulated miRNAs were identified in the La vs. Eg group (Figure 4), potentially regulating 16,601 target genes. In the PP vs. La, 24 down-regulated and 28 up-regulated miRNAs were found that potentially target 9764 target genes. A total of 37 down-regulated and 38 up-regulated miRNAs were found in Pu vs. PP, which could potentially regulate 12,632 genes. A total of 30 down-regulated and 70 up-regulated miRNAs may regulate 14,123 genes in Ad vs. Pu. However, only 17,349 genes were recorded in the genome of *M. separata* (NCBI accession number: GCA_029852925.1) [39]. Thus, miRNAs are involved in nearly every physiological process and are crucial for the development of animals [3]. It is challenging to filter several key miRNAs that regulate specific metabolic or signaling pathways, as multiple miRNAs can regulate a single mRNA, and a single miRNA targets hundreds of mRNAs [24,56,61].

Based on the KEGG enrichment analyses of genes targeted by DEmiRNAs, it is interesting that the pentose and glucuronate interconversion pathway was significantly enriched in the La/Eg, PP/La, Pu/PP, and Ad/Pu comparison groups. Over twenty *UDP-glucuronosyltransferase* (*UGT*) genes were found in this pathway. UGTs are well known for their roles in the detoxification of insecticides and xenobiotic metabolism, as well as their functions in olfaction and reproduction [62,63,64]. The temporal expression patterns of UGTs and miRNAs targeting UGTs should be further investigated in future studies.

Highly expressed miRNAs, particularly well-characterized miRNAs, have attracted significant attention from researchers. *miR-2* was found to be highly expressed in eggs, 3rd instar larvae, and pre-pupae, while being expressed at lower levels in pupae and adults (Figure 5). *miR-2a-3p* impressed *Tre-2* and *PAGM* of *Nilaparvata lugens* in the chitin biosynthesis pathway. Feeding with *miR-2a-3p* agomir caused reduced chitin content, molting defects, and a decreased survival rate [65]. The *miR-2* family (*miR-2* and *miR-13*) targets *awd* and *fringe fng* in the notch pathway, thereby regulating wing morphogenesis in *B. mori* [66]. This family also regulates oogenesis in *Locusta migratoria* by targeting genes in the *Notch* pathway [19]. Additionally, the *miR-2* family of *Blattella germanica* targets the juvenile hormone-responsive gene *Kr-h1* to regulate metamorphosis [15]. In *Diaphorina citri*, *miR-2* binds to *Kr-h1* to regulate ovarian development [67]. However, in the current study, *miR-2* targets the 3′ UTR of *cyp306a1*, a gene that encodes one of the enzymes in the 20E biosynthesis pathway, and *EcR*. EcR forms a heterodimer with ultraspiracle (USP) to respond to 20E, regulating various life processes, including metamorphosis [68,69]. When *H. armigera* larvae were fed an artificial diet containing *let-7*, which targets *EcR*, increased mortality and development defects resulted [70]. The low level of *miR-2* in the pupal and adult stages of *M. separata* may partially explain the high level of 20E during these two stages.

*miR-184* from *M. separata* was highly expressed in eggs and exhibited lower expression levels in adults (Figure 5). *miR-184* mimics affected the life history traits of *Sitobion avenae* [71]. In pea aphid, miR-184 was involved in the defense against bacterial infection [72]. In *Laodelphax striatellus*, *miR-184-3p* inhibited the expression of *Ken* and *E78*, promoting the accumulation of rice black-streaked dwarf virus (RBSDV) [73,74]. Additionally, in *Spodoptera exigua*, *miR-184* regulated the replication and infection of *Autographa californica* multiple nucleopolyhedrovirus [75]. In the current study, *miR-184* was predicted to target five *UGTs* and three *GSTs*, likely involved in detoxification processes and insect defense mechanisms.

*miR-305* displayed diverse functions in regulating target genes. The increase in *miR-305* promoted the impairment of locomotor activity and the age-dependent accumulation of poly-ubiquitinated protein aggregates in the muscles of aged *Drosophila melanogaster* [76]. The increase in *miR-305* from *B. dorsalis* binds to the 3′UTR regions of *GLIS2* in the insulin signaling pathway [21]. *Sugarbabe*, the *GLIS2* homolog, regulates insulin expression to mobilized resource substances [77]. Overexpression of *miR-305-5p* causes eclosion failure of *Panonychus citri*, which likely plays a role in deutonymph–adult transition [78]. In addition, *ame-miR-305-5p* knockdown significantly changes gene expression in the brain of *Apis mellifera*, which is associated with labor division [79]. In the current study, miR-305 targets 13 cuticle protein genes of *M. separata*, which is potentially used to interfere with cuticle formation.

*miR-279* in *Drosophila* has been proven to regulate circadian behavior through the JAK/STAT circuit [80], and the *miR-279/996* cluster in *Drosophila* repressed receptor tyrosine kinase signaling to determine cell fates in their eyes [81]. *miR-279c-5p* in *Solenopsis invicta* targets the insulin synthesis pathway to control the labor division of worker ants [82]. In the current study, *miR-279* from *M. separata* was predicted to target two glutaryl-CoA dehydrogenase genes, which are involved in lysine degradation, tryptophan metabolism, and fatty acid degradation.

Novel407 in *M. separata* was predicted to regulate *glutamine synthetase*, *voltage-dependent calcium channel L type α-1D*, three *adenylate cyclase 8* genes, and *homer*. These genes belong to the glutamatergic synapse pathway. Novel546 was predicted to target pyruvate kinase, phosphoglucomutase, and 2,3-bisphosphoglycerate 3-phosphatase, all of which belong to the glycolysis/gluconeogenesis pathway.

## 5. Conclusions

In summary, we comprehensively analyzed the miRNA developmental patterns of *M. separata*. A total of 40 known miRNAs and 360 novel miRNAs were identified. Most miRNAs were highly expressed in eggs or in eggs and pupae. DEmiRNAs targeted the majority of genes, resulting in a complex miRNA–mRNA regulation mode. Our findings will be helpful in better understanding the regulation of the developmental process in *M. separata* at a post-transcriptional level.

## Data Availability

The clean sequence data reported in this paper were deposited in the Genome Sequence Archive in the National Genomics Data Center, China National Center for Bioinformation/Beijing Institute of Genomics, Chinese Academy of Sciences (GCA bioproject number: PRJCA033466) that are publicly accessible at https://ngdc.cncb.ac.cn/gsa (accessed on 15 December 2024).

## Supplementary Materials

The following supporting information can be downloaded at https://www.mdpi.com/article/10.3390/genes16020234/s1. Table S1: Primers of miRNA and miRNA biosynthesis core genes for RT-qPCR. Table S2: Quality analyses of small RNA-seq data and the miRNA reads mapping to reference genome of *M. separata*. Table S3: Overview of small RNA sequencing data from fifteen *M. separata* libraries. Table S4: Known miRNAs from *M. separata*. Table S5: Summary of differentially expressed analyses of miRNAs from *M. separata*. Figure S1. Heatmap of the expression miRNAs from *M. saparata* in various developmental stages.

## Abbreviations

The following abbreviations are used in this manuscript:

miRNA	MicroRNA
mRNA	Messenger RNA
3′ UTR	Three prime untranslated region
5′ UTR	Five prime untranslated region
CDS	Coding sequences
Ago1	Argonaute 1
miRISC	miRNA-induced silence complex
KEGG	Kyoto encyclopedia of genes and genomes
DEmiRs	Differential expression analysis of miRNAs
PCA	Principle component analysis
*Tre-2*	*Trehalose 2*
*PAGM*	*phosphoacetylglucosamine mutase*
*awd*	*abnormal wing disc*
*fng*	*fringe*
*EcR*	*E**cdysone receptor*
*E78*	*Neuron-specific ecdysone-induced protein 78*
*UGTs*	*UDP-glucuronosyltransferases*
*GSTs*	*glutathione S-transferases*
