# Supplementary material for: Developmental Expression Patterns of miRNA in Mythimna separata Walker (Lepidotera: Noctuidae)
Source: Genes (Basel). 2025 Feb 19;16(2):234. doi: 10.3390/genes16020234 (PMC11855462; doi:10.3390/genes16020234)
Supplement: Supplementary file 1 [file genes-16-00234-s001.zip › genes-3445724-supplementary/Table S1.pdf]

Table S1 Primers of miRNA and miRNA biosynthesis core genes for RT-qPCR

Name	Primer sequence (5´-3´)
miR-2	TCACAGCCAGCTTTGATGAGC
miR-184	TGGACGGAGAACTGATAAGGGC
miR-305	ATTGTACTTCATCAGGTGCTCTGG
miR-279	TGACTAGATCCACACTCATCCA
Novel407	TCGGTGAATGCCAACGGATTTC
Novel546	CTTCGCATTCCTTCTCGCACC
U6	CGCAAAATCGTGAAGCGTTCC
Pasha	F: CTCGCAGGCCATACTACAGG
	R: CAGCTTCCGCATCTCATTGC
Dicer1	F: AGATGGACCTGGCTGAGGAT
	R: GTCGTACATCTCATCGCCGT
Ago1	F: GGACAGCAAGCATTGCCAAA
	R: CTCTGGGGTGCAAAACAAGC
