# Supplementary figures and images for: Developmental Expression Patterns of miRNA in Mythimna separata Walker (Lepidotera: Noctuidae)
Source: Genes (Basel). 2025 Feb 19;16(2):234. doi: 10.3390/genes16020234 (PMC11855462; doi:10.3390/genes16020234)

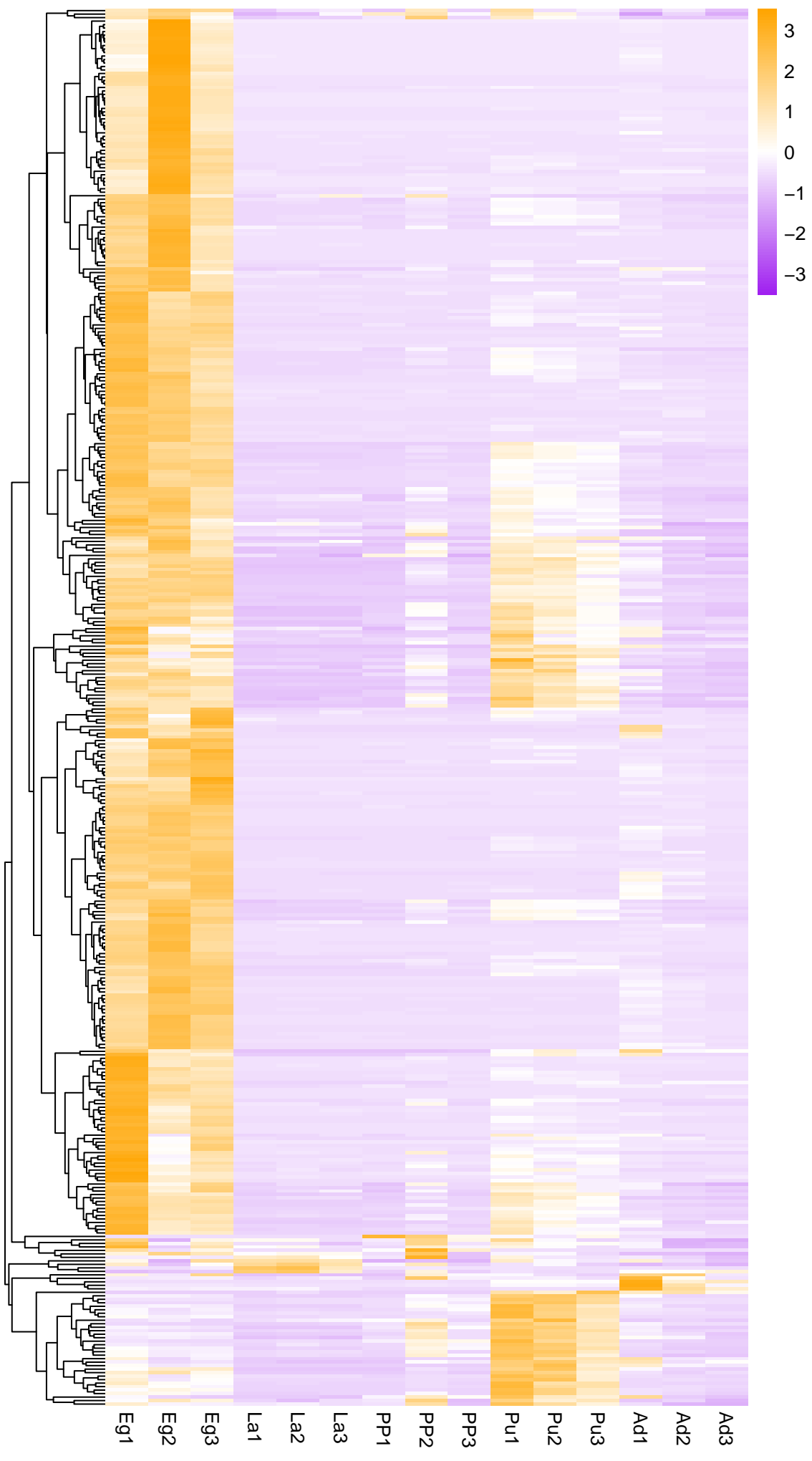

Supplement: Supplementary file 1 [file genes-16-00234-s001.zip › genes-3445724-supplementary/Fig.S1.pdf]
